# The Need for Standardized Data Collection to Improve Harmonization and Pooling of Information About Modifiable Risk Factors for Alzheimer’s Diseases in Italian Clinical Studies: A Systematic Review

**DOI:** 10.3390/geriatrics11020038

**Published:** 2026-03-31

**Authors:** Patrizio Allegra, Manuela Lodico, Claudia Migliazzo, Domenico Tarantino, Tommaso Piccoli, Nicola Vanacore, Giuseppe Salemi, Laura Maniscalco, Domenica Matranga

**Affiliations:** 1 Department of Health Promotion, Mother and Child Care, Internal Medicine and Medical Specialties—PROMISE, University of Palermo, 90127 Palermo, Italydomenica.matranga@unipa.it (D.M.); 2 Department of Diagnostic, Interventional and Stroke Radiology, UOC Neurology, AOUP “P. Giaccone”, 90127 Palermo, Italygiuseppe.salemi@unipa.it (G.S.); 3 Department of Biomedicine, Neuroscience and Advanced Diagnostics (BIND), University of Palermo, 90127 Palermo, Italy; 4 National Center for Disease Prevention and Health Promotion, Italian National Institute of Health, 00161 Rome, Italy

**Keywords:** Alzheimer disease, harmonization, obesity, physical activity, diabetes, depression

## Abstract

Background/Objectives: At the international level, harmonized networks of dementia clinical studies are available, but Italian participation remains limited. This systematic review aims to define harmonization rules to facilitate the inclusion of Italian clinical studies in existing networks and to propose standardized data collection methods to enable comparison of the study results. Methods: A systematic review was conducted (January 2019–December 2024) to identify Italian clinical studies evaluating Alzheimer’s disease and other dementias as outcomes. Eight modifiable risk factors were extracted: BMI, arterial hypertension, diabetes, dietary patterns, alcohol consumption, smoking habits, depressive symptomatology, and physical activity. WHO definitions and internationally accepted criteria were used as reference standards. Variable harmonization potential was assessed using the DataSHaPER methodology and classified as complete, partial, or impossible, considering information loss across studies. Results: Of 365 records identified, 18 studies met the inclusion criteria. Obesity assessed via BMI showed the highest harmonization potential (44% complete, 33% partial), along with dietary habits measured by food frequency questionnaires (44% complete). Diabetes and physical inactivity followed (33% complete), assessed through fasting glucose or pharmacological treatment and the IPAQ, respectively. Smoking habits classified as current, former, or never smokers were reported in 28% of studies. Depression (assessed by GDS or CES-D) and hypertension (blood pressure measurement or antihypertensive treatment) showed complete harmonization in only 22% of studies. Conclusions: Italian studies show substantial limitations in the harmonization of modifiable risk factor data for Alzheimer’s disease, mainly due to heterogeneous and non-standardized data collection methods, highlighting the need for uniform research protocols.

## 1. Introduction

Alzheimer’s disease (AD) and other dementias are the leading cause of cognitive decline, disability, or premature death among chronic diseases in the elderly. They affect over 57 [1] million people worldwide, representing a significant economic and social burden. In Europe (EU27) [2], the estimated number of people living with dementia is nearly 8 million. In Italy, the National Institute of Health (ISS) has estimated that about 1,200,000 people live with dementia, with approximately 600,000 of them suffering from AD [3].

Up to 53.7% of all cases of dementia are assumed to be due to Alzheimer disease (AD), while 15.8% are considered to be due to vascular dementia (VaD) [4].

The Lancet Commission has identified 14 modifiable risk factors for dementia, which are estimated to account for 40% of cases worldwide: low education, hypertension, hearing impairment, smoking, midlife obesity, depression, physical inactivity, diabetes, social isolation, excessive alcohol consumption, traumatic brain injury, air pollution, untreated visual impairment, and high LDL levels [1].

Modifiable risk factors, in addition to non-modifiable risk factors such as plasma levels, cerebrospinal fluid biomarkers, amyloid PET imaging, the presence of ApoE4 homozygosity, and the pharmacological treatment, are acknowledged as determinants capable of predicting disease progression.

Research in this field has produced numerous longitudinal studies aimed at investigating the progression of AD and other dementias in relation to social, environmental, and lifestyle factors, as well as genetic and pharmacological determinants.

At the international level, networks of harmonized dementia cohort studies have been developed to pursue essential goals such as facilitating clinical trial recruitment, monitoring temporal trends in dementia incidence, identifying risk factors, improving early diagnosis, and exploring targeted research questions.

The synthesis of the results of these studies provides an invaluable source of high-quality information; however, its reliability depends not only on the quantity and quality of the underlying data, but above all on the compatibility between the studies. This latter aspect implies that a “valid” synthesis of information is only possible if the collection and recording of data and information are carried out in a sufficiently similar manner across the different studies. Such synthesis requires considering only those studies that are sufficiently harmonizable with respect to certain key variables, such as major social, environmental, lifestyle, genetic, health, and pharmacological factors.

One of the most recognized international platforms is the Global Alzheimer’s Association Interactive Network (GAAIN) [5] study, a global network that promotes data sharing and collaboration among researchers to accelerate understanding and treatment of Alzheimer’s disease by creating large cohorts through the aggregation of existing Alzheimer’s disease cohorts.

Another notable network is CONCORD-AD [6], a multicenter collaboration that integrates data from seven clinical cohorts across Australia, Europe, and North America to support robust research into Alzheimer’s disease and improve clinical trial methodology.

Although Italian participation in such networks has been minimal to date, there are some notable exceptions. Italy is represented within GAAIN through I-ADNI (Italian Alzheimer’s Disease Neuroimaging Initiative) [7], which focuses on validating neuroimaging biomarkers for early diagnosis, and WMH-AD (White Matter Hyperintensities in Alzheimer’s Disease), which studies the impact of cerebral white matter changes on cognitive decline. Furthermore, Italian institutions are actively involved in the European project CONFORTage (Community-based, Integrated and People-Centric prediction, monitoring and personalized recommendations for prevention and relief of dementia and frailty) which aims to revolutionize care models for dementia and frailty by promoting integrated, community-based, and personalized healthcare strategies tailored to aging populations.

To enhance the presence of Italian cohorts within these global networks, our study sets out to establish harmonization guidelines, specifically addressing modifiable risk factors. To this aim, we conducted a systematic review of the Italian studies from 2019 to 31 December 2024 on patients with dementia, particularly AD, with the aim to extract the adopted definitions of the included modifiable risk factors. The degree of compatibility and the possibility of harmonization were established based on globally accepted and recognized definitions of modifiable risk factors by the scientific community.

This study proposes a method for data collection that can support the harmonization process and that can also be transferred to non-modifiable risk factors of AD, in order to facilitate the comparison and pooling of the findings of different clinical studies.

## 2. Materials and Methods

### 2.1. Study and Participants

We performed a systematic review and reported our findings according to the Preferred Reporting Items for Systematic Reviews and Meta-Analyses (PRISMA) guidelines [8]. Our review was registered on the International Prospective Register of Systematic Reviews (PROSPERO; 1089977).

### 2.2. Data Sources and Search Strategy

A systematic search was conducted in MEDLINE (via PubMed), Embase (via Ovid), and Scopus to identify relevant studies published between January 2019 and December 2024. The search was designed to identify clinical studies examining AD or other forms of dementia in relation to modifiable risk factors in the Italian population. Studies on data from Healthcare Utilization databases were excluded.

The database search was performed using the following search string:

<<“cognitive impairment”[All Fields] OR “dementia”[MeSH Terms] OR “dementia”[TIAB] OR “dementias”[TIAB] OR “dementia’s”[TIAB] OR “alzheimer’s”[TIAB] OR “alzheimer disease”[MeSH Terms] OR (“alzheimer”[TIAB] AND “disease”[TIAB]) OR “alzheimer disease”[TIAB] OR “alzheimer”[TIAB] OR “alzheimers”[TIAB] OR “alzheimer s”[TIAB] OR “alzheimers’s”[TIAB]) AND ((cohort studies[mesh:noexp] OR longitudinal studies[mesh:noexp] OR follow-up studies[mesh:noexp] OR prospective studies[mesh:noexp] OR retrospective studies[mesh:noexp] OR cohort[TIAB] OR longitudinal[TIAB] OR prospective[TIAB] OR retrospective[TIAB]) OR (“Case-Control Studies” [Mesh:noexp] OR “Control Groups” [Mesh:noexp] OR (case[TIAB] AND control[TIAB]) OR (cases[TIAB] AND controls[TIAB]) OR (cases[TIAB] AND controlled[TIAB]) OR (case[TIAB] AND comparison*[TIAB]) OR (cases[TIAB] AND comparison*[TIAB]) OR “control group” [TIAB] OR “control groups” [TIAB]) OR (“observational study”[Publication Type] OR “observational studies as topic”[MeSH Terms] OR “observational study”[All Fields])) AND (Italy [TIAB] OR Italian [TIAB]>>.

The reference list of studies selected for inclusion and published systematic reviews on the same topic were also screened for studies that met our inclusion criteria.

Screening by title and abstract was independently performed by three reviewers (PA, ML and CM) using Rayyan software (version 1.4.3) [9], which allowed blinding of reviewers to one another’s decisions during the screening phase. Disagreements were resolved through discussion among the three authors and, if necessary, by consultation with a senior reviewer (DM). The same procedure was applied for full-text screening.

### 2.3. Eligibility Criteria

We included: (1) studies conducted in Italian populations; (2) assessment of AD or other dementia types as the outcome diagnosed through several criteria as: Clinical Dementia Rating (CDR > 4), Diagnostic and Statistical Manual of Mental Disorders, Fourth Edition, Text Revision (DSM-IV-TR), Mild Cognitive Impairment (MCI) or Subjective Cognitive Decline (SCD) test, Short Portable Mental Status Questionnaire (SPMSQ), Mini-Mental State Examination (MMSE), Trail Making Test, Early-Onset Alzheimer’s Disease (EOAD), Early-Onset Frontotemporal Dementia (EOFTD), Diagnostic and Statistical Manual of Mental Disorders, 3rd Edition, Revised (DSM III-R), National Institute of Neurological and Communicative Disorders and Stroke and the Alzheimer’s Disease and Related Disorders Association (NINCDS-ADRDA); (3) evaluation of at least one modifiable risk factor among body mass index (BMI), arterial hypertension, diabetes, dietary patterns, alcohol consumption, depressive symptomatology, and physical activity; (4) observational cohort study design; (5) including at least five exposed participants in each group; (6) published in peer-reviewed journals; (7) written in English; (8) full-text availability.

We excluded: (1) book chapters, reviews, letters, case reports or conference abstracts; (2) studies not providing enough data to calculate the risk estimates; (3) studies in which exposure did not account for at least one modifiable risk factor; (4) studies on animal models.

### 2.4. Harmonization Approach

The extracted data from each study regarded publication year, region, study design, sample size, in addition to retrieving information about the following eight variables: BMI, arterial hypertension, diabetes mellitus, dietary patterns, alcohol consumption, depressive symptomatology, and physical activity levels. For each study, the measurement methods, the units of measurement, the assessment tools, and categorizations used for each variable of interest were recorded in detail. Afterwards, we identified potentially equivalent variables, a crucial step in the data harmonization process. As noted, the value of harmonized data fundamentally depends on the quantity and quality of the original data collected [10].

A significant challenge in this process is determining data compatibility across studies. The feasibility of harmonization depends on multiple factors, including the specific wording of questions, coding methods for ambiguous responses, treatment of missing data, and the derivation processes used to construct variables after data collection [11].

After the systematic identification of variables, we thoroughly documented different lexical formulation of these variables across the included studies. We then searched for standardized definitions among globally accepted and recognized definitions of modifiable risk factors by the scientific community.

For data harmonization, the WHO-recommended internationally recognized definitions and measurement protocols were adopted, as they enable meaningful comparisons across studies and contribute to the development of a more consistent global health evidence base. The following definitions and classifications were used to standardize data collection on modifiable risk factors:

BMI. Body Mass Index (BMI) is a simple measure of the weight-to-height ratio commonly used to classify overweight and obese adults. It is calculated by dividing weight in kilograms by the square of height in meters (kg/m^2^).

WHO Classification: Underweight: BMI < 18.5; Normal weight: BMI 18.5–24.9; Overweight: BMI 25.0–29.9; Obesity Class I: BMI 30.0–34.9; Obesity Class II: BMI 35.0–39.9; Obesity Class III: BMI ≥ 40.0 [12].

Smoking Status. Tobacco use refers to the consumption of tobacco products, mainly cigarettes, which contain nicotine and other harmful chemicals. It is one of the leading preventable causes of disease and death. WHO Classification:

- Non-smoker: A person who reports having smoked fewer than 100 cigarettes (5 packs of 20) in their lifetime and is not currently a smoker.
- Former smoker: A person who reports having smoked at least 100 cigarettes (5 packs of 20) in their lifetime, is not a smoker at the time of interview, and has quit smoking for more than 6 months.
- Current smoker: A person who reports having smoked at least 100 cigarettes (5 packs of 20) in their lifetime and is a smoker at the time of an interview or has quit smoking for less than 6 months [13].

Depression. Geriatric Depression Scale (GDS) is the gold standard for assessing depression in older adults, with the advantage of not including somatic symptoms that could be confused with physical problems typical of advanced age. Center for Epidemiologic Studies Depression Scale (CES-D) is widely used in population epidemiological studies and is particularly useful for large-scale screening.

Selection criteria based on target population:

- GDS: For elderly populations (≥65 years) [14];
- CES-D: For general population studies [15].

Physical Activity. International Physical Activity Questionnaire (IPAQ) is a validated and widely used instrument to assess physical activity levels among populations and can be used for both surveillance studies and clinical research [16].

Diabetes Diagnosis. For diabetes diagnosis, there are specific criteria used internationally. Here are the main diagnostic methods [17]:

1. Fasting plasma glucose: A fasting glucose level (after at least 8 h of fasting) equal to or greater than 126 mg/dL (7.0 mmol/L) is indicative of diabetes.
2. Random plasma glucose: A random glucose level equal to or greater than 200 mg/dL (11.1 mmol/L) in the presence of classic symptoms of hyperglycemia or hyperglycemic crisis (polyuria, polydipsia, unexplained weight loss) is a diagnostic of diabetes.
3. Oral Glucose Tolerance Test (OGTT): A glucose level equal to or greater than 200 mg/dL (11.1 mmol/L) two hours after ingestion of 75 g of glucose is indicative of diabetes.
4. Glycated hemoglobin (HbA1c): An HbA1c value equal to or greater than 6.5% (48 mmol/mol) is considered diagnostic for diabetes.

Hypertension Diagnosis. Hypertension is diagnosed based on Blood Pressure Assessment (BPA) values, when repeated measurements taken on different days show a systolic blood pressure ≥ 140 mmHg and/or a diastolic blood pressure ≥ 90 mmHg [18].

Diet. Food Frequency Questionnaire (FFQ) and Short FFQ are dietary assessment questionnaires administered in studies to evaluate dietary patterns and nutritional intake [19].

Alcohol Consumption. Alcohol consumption is measured in alcohol units. One alcohol unit corresponds to 12 g of ethanol, contained in:

- One can of beer (330 mL);
- One glass of wine (125 mL);
- One shot of liquor (40 mL).

These are at the typical alcohol concentrations of these beverages.

Currently, according to guidelines from INRAN (National Institute for Food and Nutrition Research), CDC, and other health institutions, threshold levels are based on the average alcohol units consumed per day:

- Moderate consumption threshold for men = 2 alcohol units per day;
- Moderate consumption threshold for women = 1 alcohol unit per day.

Average consumption above these quantities is considered excessive, and people who habitually drink more than these amounts are defined as “heavy drinkers” [20].

For the systematic assessment of compatibility and harmonization potential among variables across the studies included, the DataSHaPER methodology (Data Schema and Harmonization Platform for Epidemiological Research) was adopted [10]. This standardized framework provides a conceptual and operational structure to determine the degree of possible harmonization between variables from distinct studies.

For each variable and its various definitions across studies, we classified the harmonization potential as “complete”, “partial”, or “impossible” based on how each study represented the variable.

1. Complete harmonization—harmonization can be classified as “complete” when the meaning and format of the questions used in the source questionnaire allow the construction of the variable exactly as defined, without loss of information.
2. Partial Harmonization—harmonization can be classified as “partial” when the questionnaire allows the construction of the variable as defined, but with an unavoidable loss of information or precision.
3. Impossible Harmonization—harmonization can be classified as “impossible” when there is no information available in the questionnaire or the information is insufficient to construct the variable as defined.

### 2.5. Quality Assessment

The methodological quality of the included studies was assessed using the Newcastle–Ottawa Scale (NOS). The original NOS was used for cohort and case–control studies, whereas a modified NOS adapted for cross-sectional studies was applied to cross-sectional designs. The NOS evaluates methodological quality across three domains (selection, comparability, and outcome/exposure assessment), assigning a star-based score to each study. Quality assessment was conducted independently by two reviewers (PA and CM), and disagreements were resolved by consensus or consultation with a senior reviewer (LM). The NOS includes eight domains assessing methodological quality, with each study assigned a total score ranging from 0 to 8 points. Studies scoring 0–3 points were classified as low quality (LQ), those scoring 4–6 points as moderate quality (MQ), and those scoring 7–8 points as high quality (HQ). For cross-sectional studies, the modified NOS comprises nine methodological domains, with a maximum score of 9 points. Based on the total score, studies were categorized as having high (7–9 stars), moderate (4–6 stars), or low risk of bias (0–3 stars).

## 3. Results

### 3.1. Study Selection

**Figure 1:**
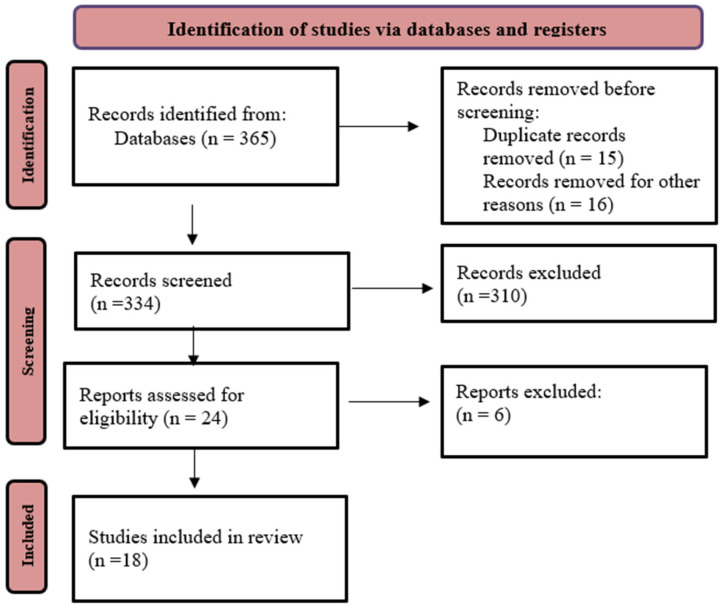
PRISMA Flow Diagram.

The initial search returned 365 records. Prior to screening, 15 duplicates were identified and were excluded, and 16 additional records were excluded due to unavailability of full text or non-English language. Following the title and abstract screening, 24 articles were selected for full-text review. After applying the inclusion criteria to the full texts, 18 studies were ultimately included in the qualitative analysis. The PRISMA flowchart can be found in Figure 1.

### 3.2. Characteristics of Included Studies

We identified 18 Italian studies [21,22,23,24,25,26,27,28,29,30,31,32,33,34,35,36,37,38] published between 2019 and 2024 that investigated the association between modifiable risk factors (such as hypertension, diabetes, obesity, physical inactivity, smoking, depression, and diet) and dementia or cognitive decline, with particular focus on AD and mild cognitive impairment (MCI). The included studies employed cross-sectional 33.3% (n = 6), longitudinal 44.4% (n = 8), and case–control designs 22.2% (n = 4). The most frequently employed method involved the use of rapid psychometric scales (e.g., SPMSQ, MMSE, CDR), which accounted for 33.3% (n = 6) of the studies; Criteria defining pre-dementia stages, specifically Mild Cognitive Impairment (MCI) and Subjective Cognitive Decline (SCD), were the second-most common category, utilized in 27.8% (n = 5) of the papers; Formal standard clinical criteria for dementia (referencing DSM-IV/V or NINCDS-ADRDA guidelines) were used in 22.2% (n = 4) of the studies; 6.7% (n = 3) of the studies focused on specific atypical or early-onset forms, such as Early-Onset Alzheimer’s Disease (EOAD) or Frontotemporal Dementia (FTD).

Sample sizes varied considerably, ranging from small clinical groups (<100) to larger population cohorts (>5000 participants) (Table S1).

The methodological quality assessment, conducted using the Newcastle–Ottawa Scale, indicated that, among cross-sectional studies, 50% were classified as having a moderate risk of bias, while the remaining 50% showed a low risk of bias. Among case–control studies, 75% were rated as having a moderate risk of bias and 25% as having a low risk of bias. Finally, all four cohort studies were assessed as having a low risk of bias (Table S2).

Within the context of this systematic review, an analysis was conducted on the compatibility and harmonizability of variables related to the main modifiable risk factors for dementia (hypertension, diabetes, obesity, smoking, physical inactivity, depression, diet). Harmonization was based on the comparability of variables reported across different studies, with the aim of including them in an integrated analysis (Table 1).

The review indicates that obesity, assessed via BMI, was comprehensively evaluated in 8 (44%) studies [21,23,24,25,26,27,28,29]. It was partially addressed in 6 (33%) studies [30,31,32,33,34,35], while in 4 (22%) studies the degree of assessment could not be determined due to missing or non-evaluable data. Obesity thus emerged as the modifiable risk factor with the highest level of complete harmonization across studies and the lowest not assessable rate (Table 2).

Dietary habits showed an equivalent rate of complete harmonization 8 (44%) studies [21,23,24,30,31,35,36,37], based on validated questionnaires such as FFQ and Mediterranean Diet Scores (MDS). However, they also exhibited a high not assessable rate (10 (55%) studies), indicating variability in methodological approaches (Table 2).

Only 6 (33%) studies [21,23,24,31,35,36] assessed physical inactivity using validated tools, such as the IPAQ, and another 6 (33%) studies [27,28,29,30,32,35] considered diabetes based on fasting glucose levels and/or ongoing pharmacological treatment. In the remaining 12 (67%) studies, harmonization of the respective modifiable factors was not feasible due to inaccessible data or insufficient documentation (Table 2).

Smoking, reported using three categories “current”, “former”, and “never smoker”, was fully harmonizable in only 5 (28%) studies [21,23,24,34,36], and partially harmonized in 3 (17%) studies [25,35,38]. In 10 (55%) studies, smoking assessment was not feasible due to the absence of data or insufficient methodological detail. These results suggest that, despite its recognition as a dementia risk, smoking is often assessed inconsistently or marginal (Table 2).

Also the harmonization attempt for both depression and hypertension yielded similar outcomes, each was analyzed according to standardized definitions in 4 (22%) studies [27,30,32,35] for the hypertension, another 4 (22%) studies [31,32,33,35] for depression, typically reported as a clinical diagnosis or via GDS for depression, and through blood pressure values and/or ongoing pharmacological treatment for hypertension. Both risk factors show the highest percentage of harmonization failure (78% impossible) compared to the other parameters analyzed.

Finally, the risk factor of alcohol consumption was considered in only 6 studies, of which 3 (17%) studies [21,23,24] achieved complete harmonization (categorized as No, Moderate, or Regular use), while the remaining 3 (17%) [30,34,35] presented partial harmonization.

Based on the data presented, depression, hypertension, and alcohol consumption appear to be the modifiable risk factors with the greatest difficulties in harmonization across the studies analyzed. In contrast, diet and obesity show a relatively high proportion of complete harmonization. Overall, impossible harmonization represents the most frequent category, accounting for 62% of all cases.

## 4. Discussion

This study represents the first systematic review specifically dedicated to assessing the harmonization potential of modifiable risk factors in Italian clinical studies on Alzheimer’s disease and other dementias. Our findings reveal significant methodological heterogeneity that drastically limits the capacity for integrating Italian data into international research networks, representing a critical missed opportunity for advancing dementia research and prevention strategies. At the international level, well-established harmonized networks such as the Global Alzheimer’s Association Interactive Network (GAAIN) and CONCORD-AD aggregate data from multiple cohorts to accelerate understanding and treatment of Alzheimer’s disease [5,6]. While Italian participation remains limited—primarily represented through I-ADNI and WMH-AD within the GAAIN—our country possesses considerable scientific heritage that, however, remains fragmented and underutilized due to lack of methodological standardization. This fragmentation is particularly concerning given that Italy has approximately 1.2 million people living with dementia, making it crucial to understand risk factors within the specific national context [3].

Our analysis of 18 Italian studies revealed alarming methodological heterogeneity in data collection and variable definition methodologies. This variability manifests as a critical issue for rigorous variable definition and successive data harmonization: only 33% of studies utilized validated tools such as IPAQ for physical inactivity assessment, while merely 28% conducted comprehensive smoking evaluations using WHO standard categories. Depression and hypertension showed the highest rate of harmonization impossibility (78%), despite being well known risk factors for dementia.

The main findings reveal considerable heterogeneity in data collection and variable definition methodologies among the included studies. Although factors such as diabetes mellitus and BMI show potential for full or partial harmonization in over 70% of studies, other crucial factors like physical inactivity, smoking, and depression proved difficult to harmonize due to lack of standardized definitions and use of non-homogeneous assessment tools. This variability represents a significant obstacle to aggregating data to obtain more robust and powerful estimates at the national level.

The lack of methodological standardization has resulted in the direct consequence of hindering meta-analyses of data collected from Italian cohorts. This represents a critical loss for several fundamental reasons. Firstly, without aggregation of multiple samples, individual studies lack the necessary statistical power to detect moderate but clinically relevant associations. Secondly, it is not possible to provide precise risk estimates for the effects of modifiable factors specific to the Italian population. Thirdly, the absence of aggregated estimates prevents the development of evidence-based prevention strategies for the national context. Pooling information from multiple studies is the only way for achieving the statistical power needed to confirm associations between risk factors and dementia, quantify population-level attributable risk, and ultimately guide prevention interventions. Our analysis demonstrates that without a priori harmonization, the value of data collected at the national level remains fragmented and underutilized.

This fragmentation is particularly problematic considering that the Lancet Commission estimates that 40% of dementia cases worldwide could be prevented or delayed by addressing 14 modifiable risk factors. Understanding the impact of these factors in the specific context of the Italian population is crucial for developing targeted and effective public health strategies, yet the preventive impact potential remains unquantifiable without harmonized data.

Despite the criticalities, our study identified areas of excellence where harmonization is already partially achievable. Obesity assessed through BMI showed 44% complete harmonization plus 33% partial harmonization, totaling 77% of potentially harmonizable studies. Dietary habits demonstrated 44% complete harmonization through FFQ and Mediterranean Diet Scores, while diabetes showed 33% complete harmonization based on standard diagnostic criteria. These results suggest that harmonization is technically feasible when internationally recognized standardized protocols are adopted.

The DataSHaPER methodology proved effective in systematically assessing the harmonization potential. This approach is transferable to biomarkers and radiologic investigations, which are increasingly used in the diagnosis of AD and other dementias, to other pathologies and risk factors, in addition to providing a methodological roadmap for future studies, and facilitating the identification of optimal definitions for complete harmonization.

The use of the DataShaper methodological application on the Italian dementia cohorts, as well as the use of rigorous and internationally validated harmonization definitions, represents one of the strengths of our study. As is well known, this approach, unlike traditional qualitative assessments, uses a rigorous metadata-based structure to quantify the accuracy of data pooling. Applying the DataSHaPER parameters allowed us to identify essential variable sets (core sets) that remained stable across different clinical contexts. This “filter” ensures that the identified “harmonization potential” is not only theoretical but also applicable in statistical practice, thanks to the rigorously validated metadata structures. Thanks to the use of internationally standardized harmonization definitions, we were able to provide a reliable roadmap for future integrative analyses in the field.

Nonetheless, it is right and necessary to acknowledge some inherent limitations in our review process. Our inclusion criteria limited the selection of articles published in English, ensuring international accessibility but also introducing the possibility of selection bias due to the exclusion of data from smaller local Italian cohorts published in national journals or in the gray literature (e.g., conference proceedings, regional reports, posters). The search timeframe was limited to the last five years (2019–2024); this choice certainly provided a very up-to-date overview of current studies but may have overlooked long-standing historical cohorts that did not publish results within this specific timeframe. Including studies with different experimental designs (cross-sectional, longitudinal, and case–control) allowed for a broader overview of the Italian scenario. However, comparing the potential for harmonization between such heterogeneous designs faced inherent challenges due to the quality of data collection, which frequently and significantly varied between retrospective and prospective settings.

## 5. Conclusions

In conclusion, this systematic review reveals significant heterogeneity in the assessment of modifiable risk factors for dementia within Italian clinical studies, which hinders data aggregation. Despite this, the study has demonstrated the feasibility and value of aggregate analyses for factors with greater harmonization potential, such as BMI and diabetes, providing risk estimates specific to the Italian population and confirming their impact.

This reinforces the urgent need for the scientific community to adopt standardized and internationally validated data collection protocols from the study design phase. Harmonizing methodologies will not only elevate the quality of national research but also facilitate the integration of Italian cohorts into global research networks, thereby accelerating progress in understanding and preventing dementia.

## Figures and Tables

**Table 1:** Definitions of modifiable risk factors for AD and other dementias adopted by 18 studies included in the systematic review.

Study	Obesity	Smoke	Depression	Physical Inactivity	Diabetes	Hypertension	Diet	Alcohol
Nicoli C. et al. 2021 [30]	BMI	Current smoker	“Life-time depression” (unrecognized questionnaire)	Self-reported	Self-reported diabetes Diabetes treatment	Self-reported hypertension Hypertension treatment	FFQ; MDs	Current alcohol user
Prinelli F. et al. 2020 [31]	BMI-WHR	Current smoker	CES-D	IPAQ	-	-	FFQ	-
Bernini S. et al. 2024 [36]	-	Current smoker Former smoker Never smoker	-	IPAQ	-	-	SFFQ	-
Giampieri F. et al. 2022 [21]	BMI	Current smoker Former smoker Never smoker	-	IPAQ	-	Self-reported hypertension	FFQ; MEDI-LITE score	No, Moderate, Regular user
Currenti W. et al. 2023 [23]	BMI	Current smoker Former smoker Never smoker	-	IPAQ	-	-	FFQ; MEDI	No, Occasional, Regular user
Currenti W. et al. 2021 [24]	BMI	Current smoker Former smoker Never smoker	-	IPAQ	Self-reported diabetes	Self-reported hypertension	FFQ	No, Moderate, Regular user
Adani G. et al. 2020 [38]	-	Ever smoker Current smoker Passive smoking exposure	-	-	-	-	-	-
Filippini T. et al. 2020 [37]	-	-	-	-	-	-	FFQ	-
Mazzoli R. et al. 2024 [22]	-	Smoker No smoker	-	-	Self-reported diabetes	Self-reported Hypertension	-	-
Soppela H. et al. 2022 [25]	BMI	Current smoker Former smoker Never smoker	-	-	Self-reported diabetes	Self-reported Hypertension	-	-
Cervellati C. et al. 2022 [26]	BMI	Current smoker	-	-	Self-reported diabetes	Self-reported Hypertension	-	-
Clark C.E. et al. 2020 [27]	BMI	Current smoker	-	-	FBG	BPA Values Hypertension treatment	-	-
Rolandi E. et al. 2020 [32]	BMI	Current smoker	GDS Depression treatment Depression History DSM-IV-TR Self-Reported Depression	Self-reported	FBG Diabetes treatment	BPA Values Hypertension treatment	-	-
Caffò AO et al. 2022 [33]	BMI-WHR	Smoker No smoker	GDS	Self-reported	-	-	unrecognized questionnaire	-
Boccardi V. et al. 2024 [28]	BMI	-	-	-	FBG Diabetes treatment	-	-	-
Noale M. et al. 2024 [34]	BMI-WHR	Current smoker Former smoker Never smoker	Self-reported depression	-	Self-reported diabetes	Self-reported Hypertension	-	heavy, light, no consumer
Franchini F. et al. 2019 [35]	BMI	Smoker No smoker	GDS	IPAQ	Self-reported diabetes Diabetes treatment	Self-reported Hypertension Hypertension treatment	MDs	low/moderate
Orlandoni P. et al. 2019 [29]	BMI	-	-	-	Self-reported diabetes FBG Diabetes treatment	-	-	-

BMI: body mass index; WHR: waist-to-hip ratio; CES-D: Center for Epidemiologic Studies Depression Scale; IPAQ: International Physical Activity Questionnaire; FFQ: Food Frequency Questionnaire; SFFQ: Semi-quantitative Food Frequency Questionnaire; GDS: Geriatric Depression Scale; DSM: Diagnostic and Statistical Manual; FBG: fasting blood glucose; BPA: blood pressure assessment.

**Table 2:** Frequency (n (%)) of papers reporting modifiable risk factors for AD and other dementias by levels of harmonization (Complete, Partial, Impossible).

Modifiable Risk Factors	Complete n (%)	Partial n (%)	Impossible n (%)	Overall
Obesity	8 (44%)	6 (33%)	4 (22%)	18 (100%)
Smoke	5 (28%)	3 (17%)	10 (55%)	18 (100%)
Depression	4 (22%)	0	14 (78%)	18 (100%)
Physical inactivity	6 (33%)	0	12 (67%)	18 (100%)
Diabetes	6 (33%)	0	12 (67%)	18 (100%)
Hypertension	4 (22%)	0	14 (78%)	18 (100%)
Diet	8 (44%)	0	10 (55%)	18 (100%)
Alcohol	3 (17%)	3 (17%)	12 (67%)	18 (100%)
Overall	44 (30%)	12 (8%)	88 (62%)	144 (100%)

## Data Availability

All data generated or analyzed during this study are included in this published article and its Supplementary Information Files. The full search strings for all databases are available in the text. Further inquiries can be directed to the corresponding author.

## Supplementary Materials

The following supporting information can be downloaded at: https://www.mdpi.com/article/10.3390/geriatrics11020038/s1, Table S1: Characteristics of the 18 reviewed studies; Table S2: Quality of evidence in cross-sectional included studies in the systematic review. Modified Newcastle-Ottawa Scale (NOS) (n = 10). References [21,22,23,24,25,26,27,28,29,30,31,32,33,34,35,36,37,38] are cited in the Supplementary Materials.

## Abbreviations

The following abbreviations are used in this manuscript:

BMI	Body mass index
WHR	waist-to-hip ratio
CES-D	Center for Epidemiologic Studies Depression Scale
IPAQ	International Physical Activity Questionnaire
FFQ	Food Frequency Questionnaire
SFFQ	Semi-quantitative Food Frequency Questionnaire
GDS	Geriatric Depression Scale
DSM	Diagnostic and Statistical Manual
FBG	fasting blood glucose
BPA	blood pressure assessment
