# Supplementary material for: The Need for Standardized Data Collection to Improve Harmonization and Pooling of Information About Modifiable Risk Factors for Alzheimer’s Diseases in Italian Clinical Studies: A Systematic Review
Source: Geriatrics (Basel). 2026 Mar 31;11(2):38. doi: 10.3390/geriatrics11020038 (PMC13116632; doi:10.3390/geriatrics11020038)
Supplement: Supplementary file 1 [file geriatrics-11-00038-s001.zip › geriatrics-4129272-supplementary.pdf]

Table S1. Supplement table: Characteristics of the 18 reviewed studies

Authors	Title	Type of study	Sample size	Mono or multicentric study	Statistics
Nicoli C. et al. 2021 [30]	The role of diet on the risk of dementia in the oldest old: The Monzino 80-plus population-based study	Cross-sectional study	1390	Monocentric	Odds Ratio
Prinelli F. et al. 2020 [31]	Exploring the relationship between Nutrition, gUT microbiota, and BRain AgINg in community-dwelling seniors: the Italian NutBrain population-based cohort study protocol	Prospective cohort study	240	Multicentric	-
Bernini S. et al. 2024 [36]	Investigating the individual and joint effects of socioeconomic status and lifestyle factors on mild cognitive impairment in older Italians living independently in the community: results from the NutBrain study	Cross-sectional study	773	Multicentric	Odds Ratio
Giampieri F. et al. 2022 [21]	Dietary Phytoestrogen Intake and Cognitive Status in Southern Italian Older Adults	Cross-sectional study	883	Monocentric	Odds Ratio
Currenti W.et al. 2023 [23]	Dietary Fats and Cognitive Status in Italian Middle-Old Adults	Cross-sectional study	883	Monocentric	Odds Ratio
Currenti W. et al. 2021 [24]	Association between Time Restricted Feeding and Cognitive Status in Older Italian Adults	Cross-sectional study	883	Monocentric	Odds Ratio
Adani G. et al. 2020 [38]	Environmental Risk Factors for Early-Onset al.zheimer's Dementia and Frontotemporal Dementia: A Case-Control Study in Northern Italy	Case-Control Study	58 cases; 54 controls	Multicentric	Odds Ratio
Filippini T. et al. 2020 [37]	Dietary Habits and Risk of Early-Onset Dementia in an Italian Case-Control Study	Case-Control Study	58 cases; 54 controls	Multicentric	Odds Ratio
Mazzoli R. et al. 2024 [22]	Atrial Fibrillation and Other Cardiovascular Factors and the Risk of Dementia: An Italian Case-Control Study	Case-Control Study	58 cases; 54 controls	Multicentric	Odds Ratio

Soppela H. et al. 2022 [25]	Modifiable potential risk factors in familial and sporadic frontotemporal dementia	Case-Control Study	1030 case; 314 control	Multicentric	Odds Ratio
Cervellati C. et al. 2022 [26]	Age, Sex, Hypertension and HDL-C Alter Serum BACE1 Activity in Cognitively Normal Subjects: Implications for Alzheimer's Disease	Cross-sectional study	175 cases; 504 controls	Multicentric	Odds Ratio
Clark C.E. et al. 2020 [27]	Systolic inter-arm blood pressure difference and risk of cognitive decline in older people: a cohort study	Cohort study	1133	Monocentric	Odds Ratio
Rolandi E. et al. 2020 [32]	Estimating the potential for dementia prevention through modifiable risk factors elimination in the real-world setting: a population-based study	Prospective cohort study	1100	Monocentric	Sub-Hazard Ratio
Caffò AO et al. 2022 [33]	The Prevalence of Amnestic and Non-Amnestic Mild Cognitive Impairment and Its Association with Different Lifestyle Factors in a South Italian Elderly Population	Retrospective cross-sectional study	839	Monocentric	Odds Ratio
Boccardi V. et al. 2024 [28]	Dysglycemia, gender, and cognitive performance in older persons living with mild cognitive impairment: findings from a cross-sectional, population-based study	Cross-sectional study	682	Monocentric	-
Noale M. et al. 2024 [34]	Undernutrition, cognitive decline and dementia: The collaborative PROMED-COG pooled cohorts study	Observational study	9071	Multicentric	-
Franchini F. et al. 2019 [35]	The LIBRA Index in Relation to Cognitive Function, Functional Independence, and Psycho-Behavioral Symptoms in a Sample of Non-Institutionalized Seniors at Risk of Dementia	Cross-sectional study	308	Multicentric	-
Orlandoni P. et al. 2019 [29]	The outcomes of long term home entral nutrition (HEN) in older patients with severe dementia	Retrospective cross-sectional study	585	Monocentric	-

Table S1. **(*continue*)**

**Authors**	**Title**	**Diagnostic criteria**	**Response rate**	**Age (years)**	**Prevalence (x 100 inhabitants)**
Nicoli C. et al. 2021 [30]	The role of diet on the risk of dementia in the oldest old: The Monzino 80-plus population-based study	DSM-IV	N/A	>80	48%
Prinelli F. et al. 2020 [31]	Exploring the relationship between Nutrition, gUT microbiota, and BRain AgINg in community-dwelling seniors: the Italian NutBrain population-based cohort study protocol	MCI	N/A	>65	N/A
Bernini S. et al. 2024 [36]	Investigating the individual and joint effects of socioeconomic status and lifestyle factors on mild cognitive impairment in older Italians living independently in the community: results from the NutBrain study	MCI	N/A	>65	24%
Giampieri F. et al. 2022 [21]	Dietary Phytoestrogen Intake and Cognitive Status in Southern Italian Older Adults	SPMSQ	85%	>50	N/A
Currenti W.et al. 2023[23]	Dietary Fats and Cognitive Status in Italian Middle-Old Adults	SPMSQ	85%	>50	N/A
Currenti W. et al. 2021 [24]	Association between Time Restricted Feeding and Cognitive Status in Older Italian Adults	SPMSQ	85%	>50	N/A
Adani G. et al. 2020 [38]	Environmental Risk Factors for Early-Onset al.zheimer's Dementia and Frontotemporal Dementia: A Case-Control Study in Northern Italy	EOAD + EOFTD	78%	<65	N/A
Filippini T. et al. 2020 [37]	Dietary Habits and Risk of Early-Onset Dementia in an Italian Case-Control Study	EOD	78%	<65	N/A

Mazzoli R. et al. 2024 [22]	Atrial Fibrillation and Other Cardiovascular Factors and the Risk of Dementia: An Italian Case-Control Study	DSM-V	N/A	65	N/A
Soppela H. et al. 2022 [25]	Modifiable potential risk factors in familial and sporadic frontotemporal dementia	FTD	N/A	<65	N/A
Cervellati C. et al. 2022 [26]	Age, Sex, Hypertension and HDL-C Alter Serum BACE1 Activity in Cognitively Normal Subjects: Implications for Alzheimer's Disease	MMSE	N/A	78	N/A
Clark C.E. et al. 2020 [27]	Systolic inter-arm blood pressure difference and risk of cognitive decline in older people: a cohort study	MMSE<br>Trail Making Test	N/A	>65	N/A
Rolandi E. et al. 2020 [32]	Estimating the potential for dementia prevention through modifiable risk factors elimination in the real-world setting: a population-based study	DSM-IV-TR	74,5%	72<br>(media)	N/A
Caffò AO et al. 2022 [33]	The Prevalence of Amnestic and Non-Amnestic Mild Cognitive Impairment and Its Association with Different Lifestyle Factors in a South Italian Elderly Population	MCI	91,5%	>60	12%
Boccardi V. et al. 2024 [28]	Dysglycemia, gender, and cognitive performance in older persons living with mild cognitive impairment: findings from a cross-sectional, population-based study	MCI	N/A	66-93	N/A
Noale M. et al. 2024 [34]	Undernutrition, cognitive decline and dementia: The collaborative PROMED-COG pooled cohorts study	DSM III-R<br>NINCDS-ADRDA	N/A	42-101	N/A
Franchini F. et al. 2019 [35]	The LIBRA Index in Relation to Cognitive Function, Functional Independence, and Psycho-Behavioral Symptoms in a Sample of Non-Institutionalized Seniors at Risk of Dementia	MCI o SCD	N/A	60-90	N/A

Orlandoni P. et al. 2019 [29]	The outcomes of long term home central nutrition (HEN) in older patients with severe dementia	Clinical Dementia Rating (CDR>4)	N/A	85,6 (media)	N/A

Table S2. (1) Quality of evidence in cross-sectional included studies in the systematic review. (2)Quality of evidence in case-control included studies in the systematic review. (3) Quality of evidence in cohort included studies in the systematic review.

**(1)**							
**Perspectives**							
**Study**	**Selection**		**Assessment of exposure and outcome**		**Confounding**		**Score**
**Cross-sectional studies**	Representativeness of the study sample	Sample size	Assessment of the exposure(s)	Assessment of the outcome(s)	Adjustment for confounder(s)	Assessment for confounder(s)	**0 to 9**
Nicoli C. et al. 2021 [30]	-	*	*	*	*	*	5
Bernini S. et al. 2024 [36]	*	*	*	*	**	*	7
Giampieri F. et al. 2022 [21]	*	*	**	*	**	*	8
Currenti W.et al. 2023 [23]	*	*	*	*	*	*	6
Currenti W. et al. 2021 [24]	*	*	**	*	**	*	8
Cervellati C. et al. 2022 [26]	*	*	**	*	**	*	8

Caffò AO et al. 2022 [33]	-	*	*	*	*	*	5
Boccardi V. et al. 2024 [28]	-	*	*	*	**	*	6
Franchini F. et al. 2019 [35]	-	-	*	*	*	*	4
Orlandoni P. et al. 2019 [29]	*	*	**	*	**	*	8

(2)

Study	Selection				Comparability	Outcome			Score
**Case-control studies**	Is the Case Definition Adequate?	Representativeness of the Cases	Selection of Controls	Definition of Controls	Comparability of Cases and Controls on the Basis of the Design or Analysis	Ascertainment of exposure	Same method of ascertainmentfor cases and controls	Non-Response rate	**0 to 8**
Adani G. et al. 2020 [38]	*	-	-	*	*	*	*	*	6
Filippini T. et al. 2020 [37]	*	-	-	*	*	*	*	*	6
Mazzoli R. et al. 2024 [22]	*	*	-	*	*	*	*	-	6
Soppela H. et al. 2022 [25]	*	*	*	*	*	*	*	-	7

(3)

Study	Selection	Comparability	Outcome	Score

**Cohort studies**	Representativeness of the exposed cohort	Selection of the non-exposed cohort	Ascertainment of exposure	Demonstration that outcome of interest was not present at start of study	Comparability of cohorts based on the design or analysis	Assessment of outcome	Was follow-up long enough for outcomes to occur?	Adequacy of follow up of cohorts	**0 to 8**
Prinelli F. et al. 2020 [31]	*	*	*	*	*	*	-	*	7
Rolandi E. et al. 2020 [32]	*	*	*	*	*	*	*	*	8
Clark C.E. et al. 2020 [27]	*	*	*	*	*	*	*	*	8
Noale M. et al. 2024 [34]	*	*	*	*	-	*	*	*	7
